# Temperature-sensitive cytoophidium assembly in *Schizosaccharomyces pombe*

**DOI:** 10.1016/j.jgg.2019.09.002

**Published:** 2019-09-20

**Authors:** Jing Zhang, Ji-Long Liu

**Affiliations:** a MRC Functional Genomics Unit, Department of Physiology, Anatomy and Genetics, University of Oxford, Oxford, OX1 3PT, United Kingdom; b School of Life Science and Technology, ShanghaiTech University, Shanghai, 201210, China

**Keywords:** CTP synthase, Cytoophidium, *Schizosaccharomyces pombe*, Heat-shock protein, Nucleoside/nucleotide metabolism, Cell biology, Yeast genetics, Cell compartmentalization

## Abstract

The metabolic enzyme CTP synthase (CTPS) is able to compartmentalize into filaments, termed cytoophidia, in a variety of organisms including bacteria, budding yeast, fission yeast, fruit flies and mammals. A previous study in budding yeast shows that the filament-forming process of CTPS is not sensitive to temperature shift. Here we study CTPS filamentation in the fission yeast *Schizosaccharomyces pombe*. To our surprise, we find that both the length and the occurrence of cytoophidia in *S. pombe* decrease upon cold shock or heat shock. The temperature-dependent changes of cytoophidia are fast and reversible. Taking advantage of yeast genetics, we demonstrate that heat-shock proteins are required for cytoophidium assembly in *S. pombe*. Temperature sensitivity of cytoophidia makes *S. pombe* an attractive model system for future investigations of this novel membraneless organelle.

## 1 Introduction

CTP synthase (CTPS) is an enzyme involved in nucleotide biosynthesis in almost all organisms. Recently multiple studies have demonstrated that CTPS forms filamentous structures termed cytoophidia in *Drosophila* (Liu, 2010), bacteria (Ingerson-Mahar et al., 2010), yeast (Noree et al., 2010; Zhang et al., 2014) and human cells (Carcamo et al., 2011; Chen et al., 2011) (reviews see Liu, 2010, Liu, 2016; Aughey and Liu, 2015). In humans and the budding yeast *Saccharomyces cerevisiae*, there are two CTPS isoforms (encoded by *CTPS1* and *CTPS2* in humans; encoded by *URA7* and *URA8* in *S. cerevisiae*), while in the fission yeast *Schizosaccharomyces pombe*, only one CTPS isoform exists and is encoded at a single locus *cts1* on chromosome I (Kim et al., 2010; Hayles et al., 2013). We have shown that among the three CTPS isoforms (CTPS isoform-A, B and C) in *Drosophila* only CTPS isoform-A is able to compartmentalize into filaments (Azzam and Liu, 2013). We also have identified that the two human CTPS isoforms, CTPS1 and CTPS2, can both compartmentalize into cytoophidia (Gou et al., 2014). The lack of CTPS redundancy in *S. pombe* gives this fission yeast an advantage as a model for the study of cytoophidia.

Noree et al. (2010) reported that CTPS and several other proteins can form filamentous structures in budding yeast. They also found that filamentation of these proteins is regulated by various environmental factors such as nutrition availability, carbon source depletion, energy status and the presence of protein synthesis inhibitors (Noree et al., 2010). In comparison with cells cultured at 30 °C, budding yeast cells grown at low temperature (0 °C) for 15 min showed no difference in the morphology and frequency of CTPS filaments (Noree et al., 2010). They concluded that filamentation of CTPS is not sensitive to temperature shift.

In *Drosophila* S2R+ cells, nutrient depletion leads to an increase in the number of cytoophidia (Aughey et al., 2014), which is parallel with the results observed in budding yeast. Similarly, cytoophidium assembly can also be induced in *Drosophila* larvae by nutrient restriction (Aughey et al., 2014). In *Drosophila* larval post-embryonic neuroblasts (pNbs), cytoophidia can be induced by nutritional restriction during the late L2 stage (Aughey et al., 2014; Tastan and Liu, 2015). Cytoophidia can also be observed in imaginal discs (eye, wing, leg and haltere) of third instar larvae fed with low-nutrient food (Aughey et al., 2014). A recent study in adult fly ovaries showed that cytoophidia increase their length upon starvation or during apoptosis (Wu and Liu, 2019).

In this study, we use the fission yeast *S. pombe* as a model system and study the response of cytoophidia to environmental stresses. Surprisingly, we find that cytoophidia are very sensitive to cold shock and heat shock. Our results suggest that cytoophidium assembly is a temperature-sensitive process in *S. pombe*, in contrast to previous observations in *S. cerevisiae*. Furthermore, we generate six heat-shock mutant strains and find that both the length and the occurrence of cytoophidia decrease significantly when one of these heat-shock proteins is disrupted. Our study highlights the importance of exploring cytoophidium assembly in multiple systems.

## 2 Results

### 2.1 Cytoophidium assembly is regulated throughout growing stages

**Fig. 1:**
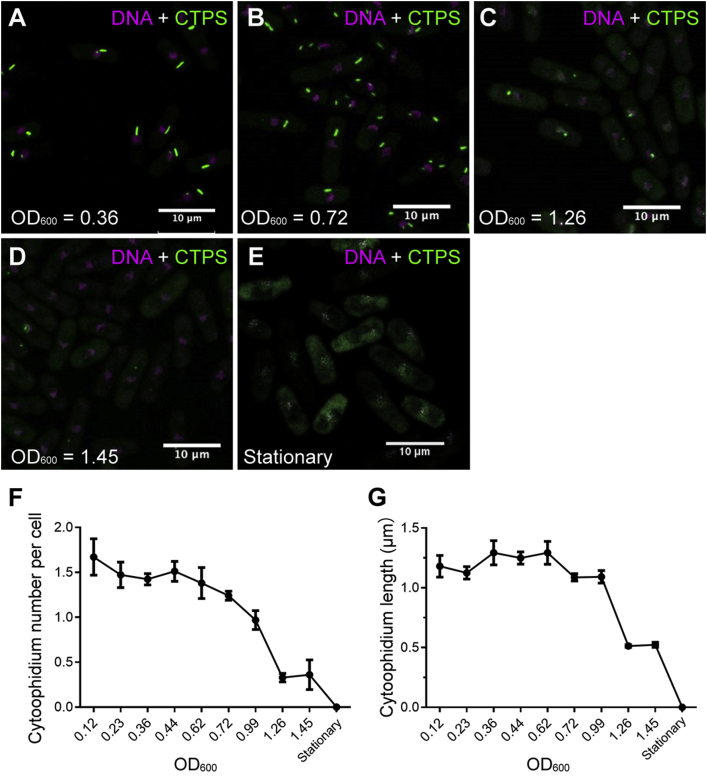
Conditions of the cell-cycle phase affect cytoophidia in *S. pombe*. **A**–**E**: CTPS distribution in *S. pombe* cells at different growth phases. **F** and **G**: Cytoophidium number per cell (**F**) and cytoophidium length (**G**) vary with growth phases. In **F** and **G**, 278–1750 cells were counted for each tested point. Error bars show SEM.

*S. pombe* cells undergo four phases such as lag phase, exponential phase, stationary phase and death phase, with differing metabolic conditions. To examine whether the different phases have an effect on cytoophidia, the CTPS-YFP expressing cells were fixed at various points in the growing stages and observed under confocal microscope. Cell growth was monitored by measuring the OD_600_ value (optical density at a wavelength of 600 nm) using the spectrometer. Each counting group contains more than 500 cells. Software ImageJ was used to measure the cytoophidium number per cell and the cytoophidium length (see details in Materials and methods). During the early-to-middle exponential phase (OD_600_ = 0.1–1.0), cytoophidia were highly abundant, being present in more than 90% of cells (Fig. 1A, B and F). As the cells grew and OD_600_ increased above 1.0, fewer cytoophidia were observed compared with the mid-exponential phase (Fig. 1C, D, and F). When cells entered the stationary phase, most of the cytoophidia disappeared, dispersing into the cytoplasm (Fig. 1E and F). This result indicates that cytoophidium regulation is closely linked to the metabolic conditions within *S. pombe* cells.

**Fig. 2:**
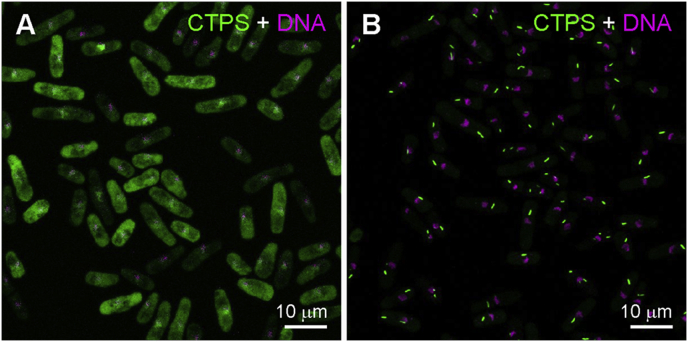
Stationary-phase-induced CTPS disassembly is reversible in *S. pombe*. **A**: Cytoophidia disperse during the stationary phase. **B**: CTPS assembles back into cytoophidia when cells are shifted to rich media and cultured for 2 h.

Next, we wanted to know if the dispersal of cytoophidia at the stationary phase is reversible. During the early-to-middle exponential phase (OD_600_ = 0.1–1.0), a high percentage of cells contained cytoophidia with a length of over 1.0 μm (Fig. 1G). When cells entered the stationary phase, the cytoophidia dispersed into the cytoplasm (Fig. 2A). When shifted to rich media and grown for another 2 h, the cells entered the exponential phase again and the number of cytoophidia increased (Fig. 2B), indicating that stationary-phase-induced cytoophidium dispersal is reversible in *S. pombe*.

### 2.2 Cytoophidia respond to a glutamine analog

**Fig. 3:**
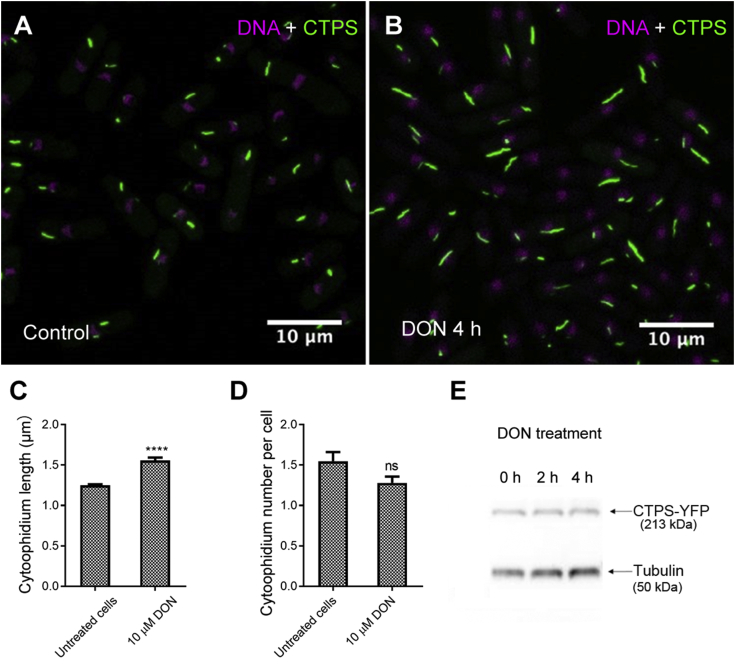
DON treatment causes an increase in the length of cytoophidia in *S. pombe*. **A** and **B**: Cells without DON treatment (**A**) or after DON treatment for 4 h (**B**). **C** and **D**: DON treatment increases cytoophidium length (**C**) but does not change the number of cytoophidia dramatically (**D**). In **C** and **D**, untreated cells, *n* = 367; DON-treated cells, *n* = 691. Error bars show SEM. Significant deference was determined by unpaired two-tailed Student’s *t*-test (****P < 0.0001). ns, not significant. **E**: Western blot of CTPS protein shows no obvious change upon DON treatment.

After treatment with the CTPS inhibitor 6-diazo-5-oxo-L-norleucine (DON), a glutamine analog, the average length of the cytoophidia was increased significantly (Fig. 3A–C), which is similar to the outcome observed in *Drosophila* and human HeLa cells (Chen et al., 2011; Aughey et al., 2014), while the cytoophidium number per cell in *S. pombe* did not changed significantly (Fig. 3D). Interestingly, the CTPS protein level was not changed after DON treatment (Fig. 3E).

### 2.3 Cold shock disassembles cytoophidia

**Fig. 4:**
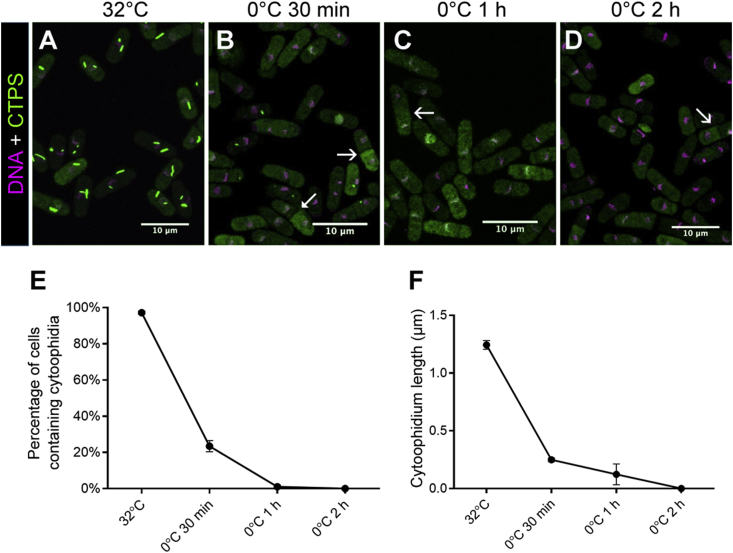
Low temperature causes decrease of cytoophidia in *S. pombe*. **A**‒**D**: When exposed to an ice-cold environment, most cytoophidia disassemble after 30 min and totally disappear after 2 h. Arrows indicate pairs of cells with asymmetric CTPS distribution. **E** and **F**: During cold shock, cytoophidia show a dramatic decrease in occurrence (**E**) and average length (**F**). In **E** and **F**, 217–940 cells were counted for each tested condition. Error bars show SEM.

When fission yeast cells were incubated at 32 °C, we found that >90% of cells at the early-to-middle exponential phase contained obvious cytoophidia (Fig. 4A and E). When cells were incubated on ice (0 °C) for 30 min, only 20% contained cytoophidia (Fig. 4B and E). Moreover, the length of the cytoophidia had decreased dramatically (Fig. 4B and F). After 1 h at 0 °C, almost all cells did not have cytoophidia, and we could only detect residual cytoophidia of very short length (Fig. 4C, E and F). After 2 h at 0 °C, CTPS showed a diffused pattern without any sign of cytoophidia (Fig. 4D–F).

We previously showed that cytoophidia are inherited asymmetrically between two daughter cells during cell cycle division (Zhang et al., 2014). One daughter cell inherits the cytoophidia from the mother cell, while the other daughter cell does not. Upon cold shock, even without cytoophidia, the asymmetric distribution of CTPS is obvious in many paired cells, with one cell having a much higher signal than the other, indicating that the protein level of CTPS is very different (Fig. 4B–D). The differential distribution of CTPS between two daughter cells is presumably achieved via asymmetric inheritance of cytoophidia.

### 2.4 Disassembly of cytoophidia induced by cold shock is fast

**Fig. 5:**
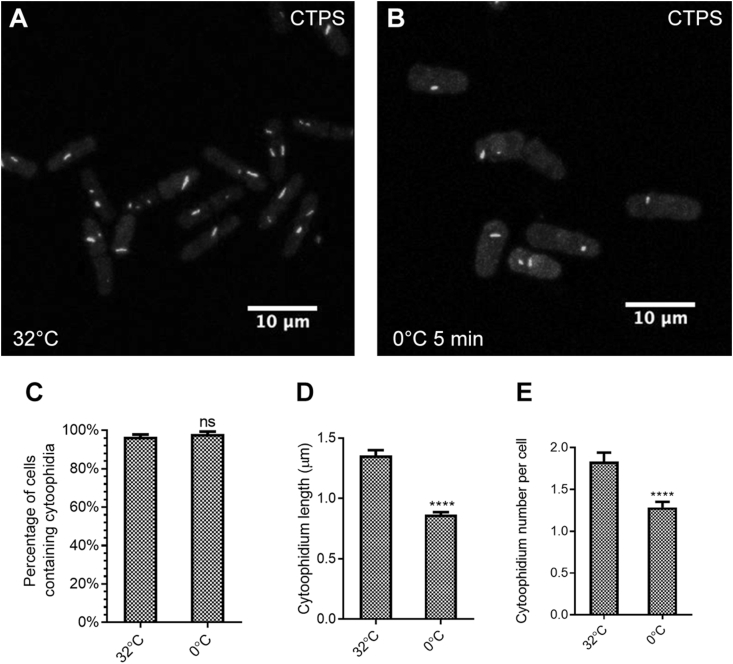
Cytoophidia respond quickly to cold stress. **A** and **B**: The length of cytoophidia is decreased significantly when *S. pombe* cells are exposed to 0 °C for 5 min. **C**: Percentage of cells containing cytoophidia is unchanged after 5 min cold shock. **D** and **E**: The average length of cytoophidia (**D**) and the number of cytoophidia per cell (**E**) have significantly decreased after 5 min cold shock, indicating that cytoophidia are extremely sensitive to temperature changes. In **C**–**E**, control (32 °C), *n* = 258 cells; cold shock (0 °C, 5min), *n* = 283 cells. Error bars show SEM. Significant difference was determined by unpaired two-tailed Student’s *t*-test (****P < 0.0001). ns, not significant.

To further investigate how quickly cold shock affects cytoophidium disassembly, we incubated fission yeast cells on ice for shorter time lengths. We found that treating cells at 0 °C for only 5 min had a profound effect on cytoophidium formation (Fig. 5A and B). Although the percentage of cells containing cytoophidia did not change significantly (Fig. 5C), we did observe that the length of cytoophidia decreased dramatically, from 1.4 μm on average to below 1 μm (Fig. 5D). The cytoophidium number per cell also significantly decreased (Fig. 5E). These results suggest that assembly of cytoophidia in *S. pombe* is extremely temperature sensitive.

### 2.5 Cold shock does not remove CTPS protein

**Fig. 6:**
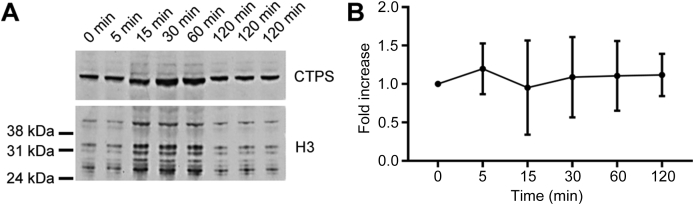
Cold shock does not remove CTPS protein. **A**: Western blot of CTPS protein under cold stress. Lanes 1–5, samples taken after cells were put on ice for 0, 5, 15, 30, 60 min, respectively. Lanes 6–8, samples taken after cells were put on ice for 2 h. **B**: Quantification of the CTPS protein levels shown in **A** indicates that CTPS protein does not disappear within 2 h of cold stress. Error bars show SD.

In *Drosophila*, the level of CTPS protein is critical for cytoophidium assembly. Overexpression of CTPS induces long and thick cytoophidia, while knockdown of CTPS disassembles cytoophidia (Azzam and Liu, 2013). To determine whether cold-shock-induced cytoophidium disassembly is due to the change of CTPS protein level, we performed Western blot of cells collected at different time points after cold-shock treatment. Anti-CTPS antibody was used for Western blot and histone H3 was used as the endogenous control. Samples were taken at 5, 15, 30, 60 and 120 min after cells were incubated on ice. We found that cold shock for up to 2 h did not remove CTPS protein (Fig. 6).

### 2.6 Cold-shock-induced cytoophidium disassembly is reversible

**Fig. 7:**
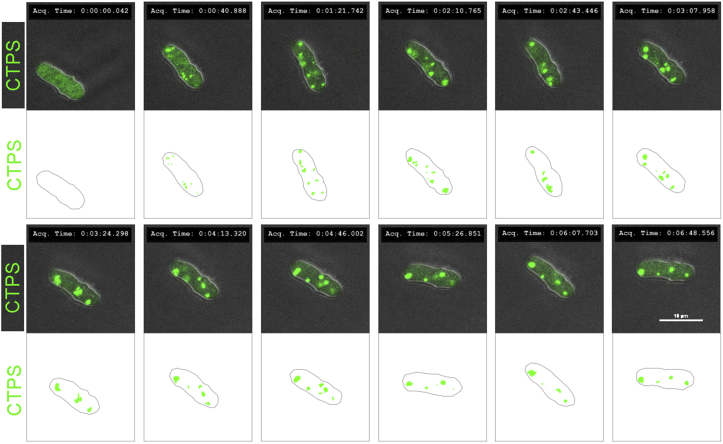
Cold-induced dispersal of cytoophidia is reversible and fast. It takes 3 min and 45 s to prepare the sample before confocal capture on the video. This time period should be added prior to the starting time point. The cells are transferred from 0 °C to 32 °C to perform the recovery experiment. Within 5 min cytoophidia (CTPS, green) start to emerge, indicating a quick response to temperature changes in *S. pombe*.

Next we asked whether cold-shock-induced cytoophidium disassembly is reversible. We incubated yeast cells at 0 °C overnight. As expected, cytoophidia disassembled completely, with CTPS showing a diffuse distribution. We then returned the yeast cells to 32 °C and observed the cells via live imaging. Within 5 min, we saw that CTPS started to form punctate structures, which grew longer over time (Fig. 7). After 30 min, we observed that many cells had cytoophidia of a size comparable to those in the control group (without cold shock) (data not shown). These results indicate that cold-shock-induced cytoophidium disassembly is reversible.

### 2.7 DON treatment does not prevent cold-shock-induced disassembly of cytoophidia

In eukaryotic cells, CTPS uses glutamine as a nitrogen donor. The glutamine analog DON binds to CTPS irreversibly. We have previously shown that DON treatment promotes cytoophidium assembly in *Drosophila* and human cells (Chen et al., 2011). DON treatment also promotes cytoophidium assembly in *S. pombe*. Treating fission yeast cells with DON for 2 h increased the cytoophidium length from 1.3 μm to 1.6 μm.

**Fig. 8:**
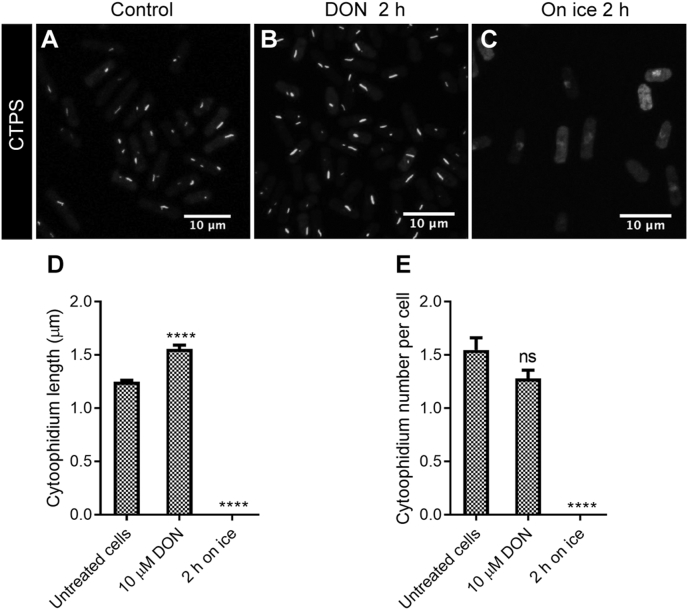
DON-induced cytoophidia can be disassembled by cold stress. **A**: Control group, with cells grown at 32 °C in YES media. **B**: Cytoophidia increase in length after cells are treated with 10 μM DON for 2 h. **C**: Cytoophidia are disassembled after cells are put on ice for 2 h. White, CTPS. **D** and **E**: Quantification of cytoophidium length (**D**) and cytoophidium number per cell (**E**) in each group. In **D** and **E**, 247–841 cells were counted for each tested group. Error bars show SEM. Significant difference was determined by one-way ANOVA (****P < 0.0001). ns, not significant.

To determine whether DON-induced cytoophidium assembly is reversible by cold-shock induction, we incubated fission yeast cells in DON for 2 h at 32 °C followed by incubating them at 0 °C for 2 h. We found that cytoophidia were no longer detectable after incubation at 0 °C (Fig. 8). These results indicate that 1) DON-induced cytoophidium assembly is reversible and 2) DON treatment cannot prevent cold-shock-induced cytoophidium disassembly.

### 2.8 Heat shock disassembles cytoophidia

**Fig. 9:**
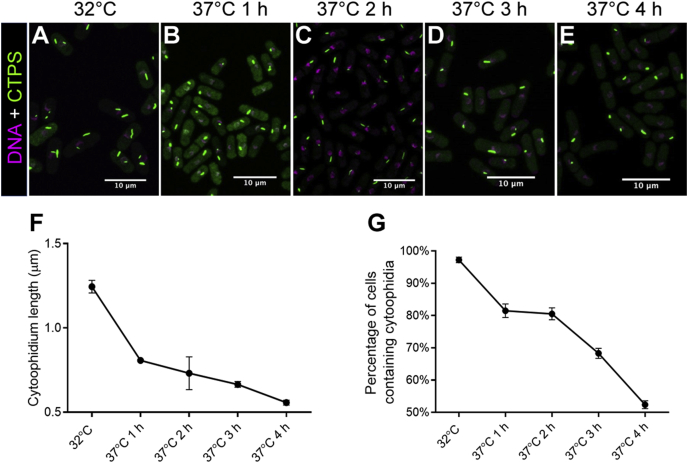
High temperature causes decrease of cytoophidia in *S. pombe*. **A**–**E**: Cytoophidia are sensitive to temperature shift from 32 °C to 37 °C. **F** and **G**: When exposed to 37 °C, both cytoophidium length (**F**) and percentage of cells containing cytoophidia (**G**) decrease. In **F** and **G**, 398–1132 cells were counted for each tested group. Error bars show SEM.

Having observed that cytoophidium assembly is sensitive to cold shock, we next asked whether heat shock affects cytoophidium assembly in fission yeast. At 32 °C, the majority of *S. pombe* cells in the early-to-middle exponential phase contain obvious cytoophidia (Fig. 9A). When exposed to a higher temperature (i.e., 37 °C or 42 °C), cytoophidia showed a decrease in both the average length and the number of cells containing cytoophidia (Fig. 9B–G).

**Fig. 10:**
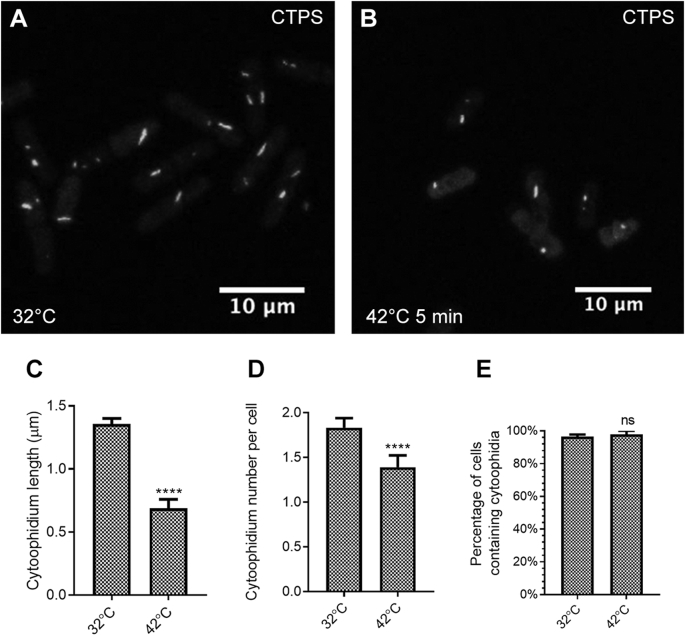
Cytoophidia respond quickly to heat stress. **A** and **B**: Cytoophidium length decreases in response to heat shock after only 5 min, comparing cells cultured at 32 °C (**A**) and 42 °C (**B**). White, CTPS. **C** and **D**: Average length of cytoophidia (**C**) and average number of cytoophidia per cell (**D**) are decreased significantly when *S. pombe* cells are exposed to 42 °C. **E**: Percentage of cells containing cytoophidia is unchanged after 5 min heat shock. In **C**–**E**, control (32 °C), *n* = 258 cells; heat shock (42 °C, 5 min), *n* = 261 cells. Error bars show SEM. Significant difference was determined by unpaired two-tailed Student’s *t*-test (****P < 0.0001). ns, not significant.

Similar to cold shock, heat shock affects cytoophidium assembly very quickly (Fig. 10A and B). Cytoophidia significantly decreased in both the length and the number of cytoophidia per cell after only 5 min of heat-shock treatment at 42 °C, compared with cells cultured at 32 °C (Fig. 10A‒D). However, the percentage of cells containing cytoophidia was unchanged after 5 min heat shock (Fig. 10E).

### 2.9 Heat-shock proteins are required for cytoophidium assembly

**Fig. 11:**
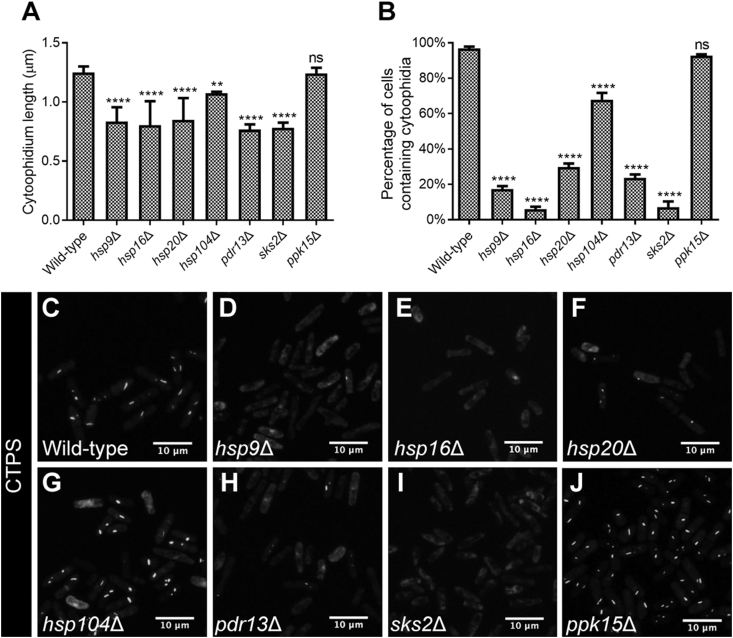
Cytoophidium morphology is affected in heat-shock protein deletion strains. **A** and **B**: Average length of cytoophidia (**A**) and percentage of cells containing cytoophidia (**B**) significantly decrease in all six heat-shock protein deletion strains tested. For each tested group, 224–722 cells were counted. Error bars show SEM. Significant difference was determined by one-way ANOVA (****P < 0.0001 and **P < 0.01). ns, not significant. **C**–**J**: Different deletion strains show different CTPS-YFP distribution patterns. *ppk15*Δ cells are used as a second control group.

Heat-shock proteins are a family of proteins produced in response to environmental stresses, especially heat-shock stress. To determine whether small heat-shock proteins are required for cytoophidium assembly, we selected several small heat-shock proteins in *S. pombe* cells for further investigation. We constructed a series of heat-shock protein deletion strains expressing CTPS-YFP, namely *hsp9*Δ, *hsp16*Δ, *hsp20*Δ, *hsp104*Δ, *pdr13*Δ and *sks2*Δ. Since *ppk15*, encoding a serine/threonine protein kinase, is not related to heat-shock proteins or heat-shock response, we used the *ppk15*Δ strain as an extra control alongside the wild-type control. We found that both the length and the occurrence of cytoophidia decreased significantly in all six heat-shock mutant strains that we investigated (Fig. 11). Our results indicate that heat-shock proteins are required for cytoophidium assembly.

## 3 Discussion

### 3.1 Comparison of cytoophidia between *S. cerevisiae* and *S. pombe*

Several studies have shown that CTPS compartmentalizes into filamentous structures in various organisms, including bacteria, fruit fly, rat and human cells (Ingerson-Mahar et al., 2010; Liu, 2010; Noree et al., 2010; Carcamo et al., 2011; Chen et al., 2011). It has also been reported that CTPS filaments can be observed in budding yeast (Noree et al., 2010, 2014; Shen et al., 2016; Zhang et al., 2018). We have previously identified that CTPS forms cytoophidia in *S. pombe* (Zhang et al., 2014; Li et al., 2018). These observations suggest that cytoophidia are evolutionarily conserved from bacteria to humans.

Both budding yeast and fission yeast are good model organisms to study cytoophidia. It has already been reported that the expression products of both *CTPS* genes (*URA7* and *URA8*) can compartmentalize into filaments in *S. cerevisiae* (Noree et al., 2010). In budding yeast, cytoophidia respond to several drugs and environment stresses (Noree et al., 2010). Here we show that cytoophidia are also sensitive to DON and stresses in fission yeast. However, there are some notable differences in cytoophidium behavior between *S. cerevisiae* and *S. pombe*.

In budding yeast, cytoophidia can be detected mostly in the stationary phase but not in the exponential phase (Noree et al., 2010; Shen et al., 2016). In *S. pombe*, we show that only exponential cells contain large numbers of cytoophidia while few or no cytoophidia can be observed during the stationary stage. Moreover, carbon deprivation induces cytoophidium assembly in budding yeast (Noree et al., 2010), while here we found that the lack of a carbon source causes the dispersal of cytoophidia in fission yeast. These opposing results suggest that the regulatory mechanism of cytoophidium assembly differs between *S. pombe* and *S. cerevisiae*.

### 3.2 The effect of DON on cytoophidium assembly

DON is an inhibitor for many glutamine-utilizing enzymes including CTPS. It has been reported that the majority of cytoophidia in human cells and *Drosophila* are an inactive form of CTPS (Aughey et al., 2014; Barry et al., 2014; Noree et al., 2014), leading to a hypothesis that cytoophidia act as a CTPS storage depot as well as a regulator of CTPS metabolic activities. When treated with DON, *S. pombe* showed an increase of the average length of cytoophidia. Similar results can be detected in human HeLa cells, *Drosophila* S2 cells and *Drosophila* tissues (Chen et al., 2011; Aughey et al., 2014), indicating that the promotion of cytoophidium assembly by DON is highly conserved.

Interestingly, although the average length of cytoophidia was increased after DON treatment, the average number of cytoophidia per cell did not change dramatically in *S. pombe*. The small cytoophidia may have fused into longer and thicker filaments, and therefore increased in filament length. This is similar to the maturation process of cytoophidia observed in mammalian culture cells (Gou et al., 2014; Chang et al., 2018; Keppeke et al., 2018).

### 3.3 Cytoophidia respond to temperature shift

Our study has revealed that cytoophidium maintenance is sensitive to temperature shift, both to cold shock and to heat shock. Given that the cytoophidium formation is growth phase-dependent in fission yeast, there is an argument that the temperature sensitivity is due to the cells switching between the exponential phase and the stationary phase upon temperature shift. The response of cytoophidia to heat or cold stress is fast (within a few minutes), while the time course of growth phase changes is slow (from a few hours to a few days). Therefore we think the dynamics of cytoophidia during temperature shift cannot be simply explained by growth phase changes.

Using yeast genetics, we have shown the involvement of heat-shock proteins, hsp9, hsp16, hsp20, hsp104, pdr13/ssz1 and sks2, in cytoophidium assembly. Among these six proteins, only hsp9 has been reported to be involved in vegetative growth metabolism under normal growing temperature conditions, while the other five proteins play roles during various stress responses.

Heat-shock proteins are a family of proteins that are produced by cells in response to exposure to stressful conditions. They were first described in relation to heat shock, but are now known to be expressed during other stresses including exposure to cold and UV light, and during wound healing or tissue remodeling (Li and Srivastava, 2004). Many members of this protein family perform a chaperone function by stabilizing new proteins to ensure correct folding or by helping to refold proteins that are damaged by the cell stress. This increase of expression is transcriptionally regulated (Wu, 1995). The dramatic upregulation of the heat-shock proteins is a key part of the heat-shock response and is induced primarily by heat-shock factor (HSF) (Wu, 1995). Heat-shock proteins are found in virtually all living organisms, from bacteria to humans.

### 3.4 Potential functions of cytoophidia in *S. pombe*

Recently, an increasing number of metabolic enzymes have been identified as having the ability to self-assemble and to form filamentous structures, including CTPS, suggesting that intracellular compartmentation of metabolic enzymes is more general than previously thought. A large-scale screen of a yeast GFP library identifies that over 20% of the examined proteins are able to from distinct intracellular structures (Narayanaswamy et al., 2009). Another screen of 40% of a budding yeast GFP strain collection shows that nine proteins form four types of filamentous structure (Noree et al., 2010). A further comprehensive screen of 4159 proteins in budding yeast reveals that at least 23 proteins have filament-forming capacity (Shen et al., 2016). Several studies demonstrate that polymerization of CTPS into cytoophidia downregulates enzymatic activity in bacteria (Barry et al., 2014), budding yeast (Noree et al., 2014), fruit flies and human cell lines (Aughey et al., 2014). Additional studies suggest that forming filaments can upregulate enzymatic activities (Strochlic et al., 2014; Chang et al., 2015; Lynch et al., 2017). Thus, cytoophidium formation seems to be a general strategy to regulate enzymatic activity. The choice of upregulation or downregulation may reflect the context of developmental stages or metabolic statuses.

This study demonstrates that cytoophidium assembly is a temperature-sensitive process in *S. pombe*. The assembly and disassembly of the cytoophidium and its kind provide a potential mechanism to maintain cellular homeostasis during development and in mediating metabolic responses (Aughey et al., 2014; Barry et al., 2014; Noree et al., 2014; Petrovska et al., 2014; Strochlic et al., 2014). Yet it is still not clear whether the disassemaembly of cytoophidia is directly or indirectly caused by the action of heat-shock proteins. More recently, we found that forming cytoophidia prolongs the half-life of CTPS in human cell lines (Sun and Liu, 2019). Further studies are needed to determine how various heat-shock proteins cooperate in the processes of assembly, maintenance and disassembly of cytoophidia.

## 4 Materials and methods

### 4.1 *S. pombe* strains

The generation of the CTPS-YFP-expressing *S. pombe* strain has been described in our previous study (Zhang et al., 2014).

### 4.2 *S. pombe* cell culture

If not mentioned, all cells were cultured at 32 °C in standard rich media (YE3S media, YES enriched with supplemwnts adenine, leucine and uracil at 100 μM) using a shaking incubator. Cell growth was monitored by OD_600_ (optical density at a wavelength of 600 nm), with a 0.1–1.5 OD_600_ value indicating exponentially growing cells and over 1.5 OD_600_ indicating stationary cells. Two wild-type *S. pombe* strains, *001 (h+; ade6-M216, leu1-32, ura4-D18)* and *002 (h-; ade6-M216, leu1-32, ura4-D18)*, were obtained from the Stephen Kearsey Laboratory (University of Oxford, UK). The *003 (h+; ade6-M216, leu1 32, ura+, cts1-yfp)* strain was used as the control unless stated otherwise.

### 4.3 Construction of heat-shock protein deleted strains

The *hsp9, hsp16, hsp20, hsp104, pdr13, sks2 and ppk15* genes were deleted from the CTPS-YFP expressing strain by PCR-based gene targeting approaches. The pFA6A-KanMX6 plasmid was used as the template plasmid. Each plasmid contained the 60-bp upstream and downstream endogenous regions of interested genes. In-fusion Cloning (Takara Clontech, USA) was utilized to integrate the 60-bp upstream and downstresm fragments in to the plasmids. The standard commercial protocol was used for the detailed manipulation of plasmid construction. The primers used in In-fusion were designed on the website and the overlaps of the primers were designed with 25 bp at least. Plasmids were verified by colony PCR and then linearized and transformed into the cts1-YFP strain. The successfully transformed strains had the replacement of interested genes with the KanMX6 cassette and then verified by G418 selection.

### 4.4 Light microscopy

Cells were cultured overnight before being fixed and scanned, and were guaranteed to be fresh and in the exponential phase. Cells were fixed with 4% paraformaldehyde in phosphate-buffered saline for 10 min and Hoechst 33342 (1 μg/mL; Thermo Fisher Scientific, USA) was used to label DNA. For live imaging, *S. pombe* cells were cultured in glass-bottomed Petri dishes with YES and kept at 32 °C. Imaging of fixed cells or time lapse for live cells was acquired using a Leica TCS SP5 II confocal microscope or Nikon A1R+ confocal microscope. Image processing and analysis were conducted using ImageJ or GraphPad Prism 7.

### 4.5 *S. pombe* whole-genome DNA purification

*S. pombe* cells were grown overnight and washed once with sterile water. Cells were re-suspended in 0.2 mL of lysis buffer (2% Triton X-100, 1% SDS, 100 mM NaCl, 10 mM Tris-HCl pH 8.0, and 1 mM EDTA pH 8.0), 0.2 mL of phenol:chloroform together with 0.5 mL of glass beads (425–600 μm; G8772, Sigma, USA), and vortexed for 10 min. Then, 0.2 mL of TE buffer (10 mM Tris-HCl pH 8.0 and 1 mM Na-EDTA pH 8.0) was added and the cells were centrifuged at 15,871×*g* for 5 min. The supernatant was transferred into a new 1.7-mL Eppendorf tube and 1 mL of 100% ethanol was added. Cells were centrifuged at 15,871×*g* for 5 min and washed with 100% ethanol once. Supernatant was discarded and the pellet was dried at room temperature. The pellet was resuspended in 50–100 μL of EB buffer.

### 4.6 Preparation of *S. pombe* total proteins

The TCA protein extraction method was used. Cells were grown to an OD_600_ of 0.2 and centrifuged at 3500×*g* for 5 min. Then, they were resuspended in 1 mL of 1.2 M sorbitol and transferred to a 2-mL Eppendorf tube before centrifugation at full speed for 30 s. The supernatant was discarded and 100 μL of 20% TCA (100 g TCA in 227 mL water) was added to resuspend the cells. Then, 0.5 mL of glass beads (425–600 μm; G8772, Sigma) was added, and the cells were put under vortex movement using Genie 2 for 10 min at 4 °C, left to stand for 1 min and put under vortex movement again for 1 min. They were vortexed again briefly after adding 900 μL of 5% TCA (25 g TCA in 227 mL water). An 800-μL extract was transferred to a new 1.7-mL Eppendorf tube and centrifuged for 10 min at 1000×*g*. The supernatant was discarded, and 250 μL of 1 × Laemmli sample buffer (62.5 mM Tris-HCl pH 6.8, 10% glycerol, 2% SDS, 5% β-mercaptoethanol, and 0.05% BPB) was added followed by resuspension (more 1 M Tris-HCl pH 8.0 could be added if solution turned yellow). After boiling for 3 min and cooling on ice, the extract was centrifuged at 1000×*g* for 10 min. Then, the supernatant was transferred to a new 1.7-mL Eppendorf tube, kept frozen at −20 °C, and thawed by boiling for 3 min every time a sample was used.

### 4.7 SDS-PAGE and Western blot

The NuPAGE MOPS System (Thermo Fisher Scientific) was used to perform the SDS-PAGE and Western blot. For antibody staining, membrane was incubated in primary antibody solution (5% milk in TBST + 1:200 antibodies; the dilutions can vary among different antibodies) overnight at 4 °C. It was washed with TBST three times and each time for 10 min (shaking), followed by incubation with secondary antibody solution (5% milk in TBST + 1:5000 antibodies) at room temperature for 1 h. SuperSignal West Pico PLUS Kit (Thermo Fisher Scientific) was used for visualization.

### 4.8 Statistical analysis

The statistical analysis and the produce of graphs were performed with Prism (v7, GraphPad, CA, USA). All data were expressed as mean ± SEM or mean±SD. Unpaired two-tailed Student’s *t*-test or one-way ANOVA was used to determine the significant differences (*P < 0.05, **P < 0.01, ***P < 0.001 and ****P < 0.0001). ns, not significant.
